# B-Cell Activating Factor Secreted by Neutrophils Is a Critical Player in Lung Inflammation to Cigarette Smoke Exposure

**DOI:** 10.3389/fimmu.2020.01622

**Published:** 2020-07-29

**Authors:** Mégane Nascimento, Sarah Huot-Marchand, Aurélie Gombault, Corinne Panek, Manon Bourinet, Manoussa Fanny, Florence Savigny, Pascal Schneider, Marc Le Bert, Bernhard Ryffel, Nicolas Riteau, Valérie F. J. Quesniaux, Isabelle Couillin

**Affiliations:** ^1^University of Orleans and CNRS, INEM-UMR7355, Orléans, France; ^2^Department of Biochemistry, University of Lausanne, Épalinges, Switzerland

**Keywords:** B cell activating factor, cigarette smoke, pulmonary inflammation, neutrophils, mice

## Abstract

Cigarette smoke (CS) is the major cause of chronic lung injuries, such as chronic obstructive pulmonary disease (COPD). In patients with severe COPD, tertiary lymphoid follicles containing B lymphocytes and B cell-activating factor (BAFF) overexpression are associated with disease severity. In addition, BAFF promotes adaptive immunity in smokers and mice chronically exposed to CS. However, the role of BAFF in the early phase of innate immunity has never been investigated. We acutely exposed C57BL/6J mice to CS and show early BAFF expression in the bronchoalveolar space and lung tissue that correlates to airway neutrophil and macrophage influx. Immunostaining analysis revealed that neutrophils are the major source of BAFF. We confirmed *in vitro* that neutrophils secrete BAFF in response to cigarette smoke extract (CSE) stimulation. Antibody-mediated neutrophil depletion significantly dampens lung inflammation to CS exposure but only partially decreases BAFF expression in lung tissue and bronchoalveolar space suggesting additional sources of BAFF. Importantly, BAFF deficient mice displayed decreased airway neutrophil recruiting chemokines and neutrophil influx while the addition of exogenous BAFF significantly enhanced this CS-induced neutrophilic inflammation. This demonstrates that BAFF is a key proinflammatory cytokine and that innate immune cells in particular neutrophils, are an unconsidered source of BAFF in early stages of CS-induced innate immunity.

## Introduction

Inflammatory lung diseases represent a major public health problem. Their incidence is constantly increasing and the predictions of the World Health Organization (WHO) are at least pessimistic over the next 20–30 years, reflecting recent changes in our society and the associated consequences on air quality and social behavior. In particular, chronic obstructive pulmonary disease (COPD) is characterized by chronic bronchitis and pulmonary emphysema and is triggered by repeated airway exposure to harmful particles mainly cigarette smoke (CS) (1). WHO projections anticipate that this chronic and progressive pathology will become the third leading cause of death by 2030. In this context, it has become a priority to increase our research efforts to better understand the cellular and molecular mechanisms involved in the pathophysiology of pulmonary inflammation in order to propose new innovative therapies.

A sustained cellular inflammation characterized by airway neutrophilic recruitment is commonly correlated with bad prognosis in chronic lung inflammation and COPD exacerbations elicited by a bacterial or viral infection (2). Paradoxically, this strong inflammatory response is associated with impaired bacterial clearance due to ineffective neutrophils which do not prevent the occurrence of infection-driven exacerbations (1). Activated neutrophils release reactive oxygen species as well as pre-formed proteases and lytic enzymes from intracytoplasmic granules, or burst into neutrophil extracellular traps (3). However, in addition to proinflammatory effects, neutrophils can display immunosuppressive potential (4, 5). The transition between proinflammatory and immunosuppressive neutrophils is highly dynamic and neutrophil plasticity may influence immune responses to environmental stress such as CS exposure (6).

COPD is also characterized by a significant increase in the number of alveolar macrophages in the airways of patients which correlates with airway obstruction and COPD severity (7). Both immune and non-immune cells such as alveolar epithelial cells participate in the inflammatory cell recruitment consecutive to CS exposure through their ability to produce chemokines and cytokines. B cell activating factor (BAFF) belongs to the tumor necrosis factor (TNF) cytokine family, shown first to play a key role in the maturation and survival of B lymphocytes and in the establishment of the humoral response (8, 9). However, recent data indicate that BAFF may also participate in the regulation of innate immune responses, particularly at the level of the respiratory mucosa (10, 11).

BAFF is mainly produced by adaptive immune cells, in particular B (12, 13) and T (14) lymphocytes as well as stromal cells especially pulmonary epithelial cells (15). Innate immune cells such as neutrophils (16), monocytes/macrophages and dendritic cells can also produce BAFF depending on the context. BAFF is expressed as a membrane-bound or soluble protein and its excessive expression leads to the development of autoimmune disorders in mice and humans (17–19). There are three BAFF receptors, BAFF receptor (BAFFR), transmembrane activator and the cyclophilin ligand interactor (TACI), and B cell maturation antigen (BCMA), all expressed by B and T lymphocytes but also by antigen presenting cells, indicating that BAFF function extends beyond that of B cell biology (15).

In addition to its important role in the production of autoantibodies and in autoimmunity, BAFF is implicated in the pathophysiology of pulmonary diseases. In particular, we showed high BAFF levels in the bronchoalveolar lavage fluid of patients with idiopathic pulmonary fibrosis (10). We also showed that genetic ablation of BAFF or BAFF neutralization significantly attenuated pulmonary fibrosis (10). Importantly, B cell BAFF expression generates a self-perpetuating loop implicated in COPD progression by promoting pulmonary B cell survival and tertiary lymphoid follicles expansion (11, 20–23).

In models of mice chronically exposed to CS, BAFF plays a major role in the generation of pulmonary antinuclear antibodies and tertiary lymphoid follicles (11, 23). In addition, BAFFR-Fc administration significantly attenuated lung inflammation and alveolar wall destruction (23). However, the role of BAFF in the development of innate immunity to cigarette smoking has never been investigated. Here, we report that acute CS exposure in mice induces BAFF expression in both the bronchoalveolar space and lungs and revealed neutrophils as the major source of BAFF during early inflammation. We also show a critical role of BAFF in acute inflammation to CS. Our results suggest that BAFF is a crucial mediator in the crosstalk between innate and adaptive immune responses in the context of CS-induced inflammation and may provide cues for new therapeutic targets for COPD and COPD exacerbations.

## Results

### Acute Cigarette Smoke Exposure Promotes BAFF Expression in Airway Recruited Neutrophils

**Figure 1:**
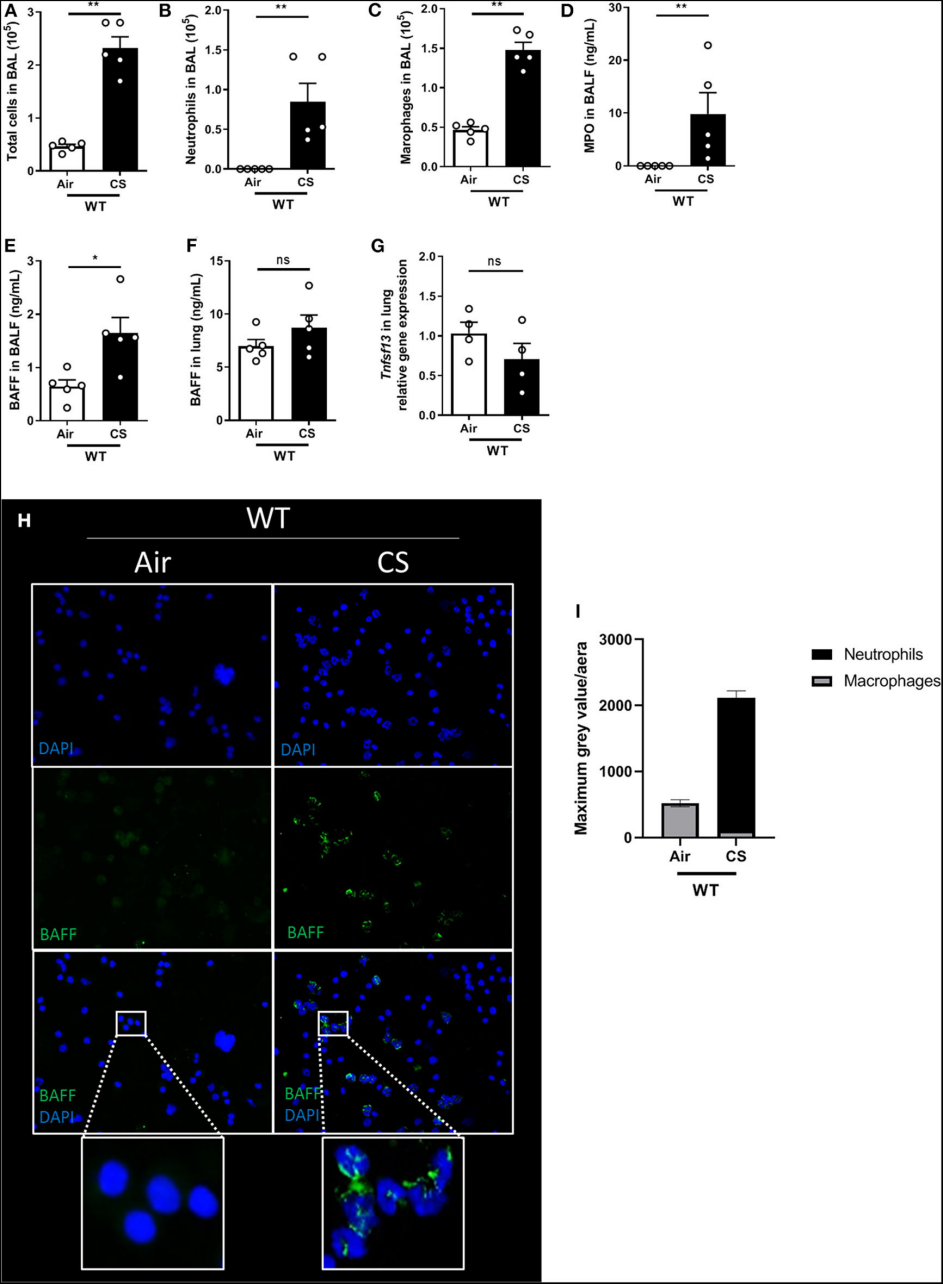
Acute CS-exposure induces BAFF expression in airways-recruited neutrophils. Mice were exposed to air (Air) or cigarette smoke (CS) three times a day for 4 days and sacrificed 16 h after the last exposure. Total cells **(A)**, neutrophils **(B)**, and macrophages **(C)** counted in BAL. MPO levels in BALF **(D)**. BAFF levels, respectively, in BALF and lung **(E,F)**. *Tnfsf13* mRNA levels (coding for APRIL) in lung homogenates **(G)**. BAFF immunostaining on BAL cells collected after air or CS exposure: BAFF shown in green and nucleus in blue (DAPI) **(H)**. Fluorescent intensity quantification **(I)**. *n* = 4–5 mice per group. Bar graphs are expressed as mean ± SEM. ns, non-significant; *p < 0.05 and **p < 0.01.

To address the role of BAFF in the development of acute lung inflammation in response to cigarette smoke (CS), C57BL/6J wild type mice (WT) were exposed to 4 cigarettes, three times a day for 4 days and sacrificed 16 h after the last exposure. WT mice exposed to CS presented significant increase in the number of total cells recruited into the bronchoalveolar lavage (BAL) (Figure 1A) and in particular neutrophils (Figure 1B) and macrophages (Figure 1C), as well as enhanced myeloperoxidase (MPO) levels (Figure 1D) in BAL fluid (BALF) which correlates with neutrophil recruitment. Interestingly, we observed enhanced amounts of BAFF in the BALF and a trend in the lung homogenates (Figures 1E,F). Of note we did not observe a significant change in *Tnfsf13* mRNA levels (as known as APRIL, another TNF family member; Figure 1G). BAL cells immunostaining assay employing BAFF specific antibody indicates that airways recruited neutrophils, identified by their multi-lobulated nucleus stained with DAPI, strongly express BAFF in response to CS (Figures 1H,I). At this time point, BAL macrophages did not seem to express high levels of BAFF, suggesting that neutrophils are the major source of BAFF produced in the bronchoalveolar space in response to acute CS-exposure.

### Neutrophil Depletion Partially Decreases BAFF Expression in Lung of CS-Exposed Mice

**Figure 2:**
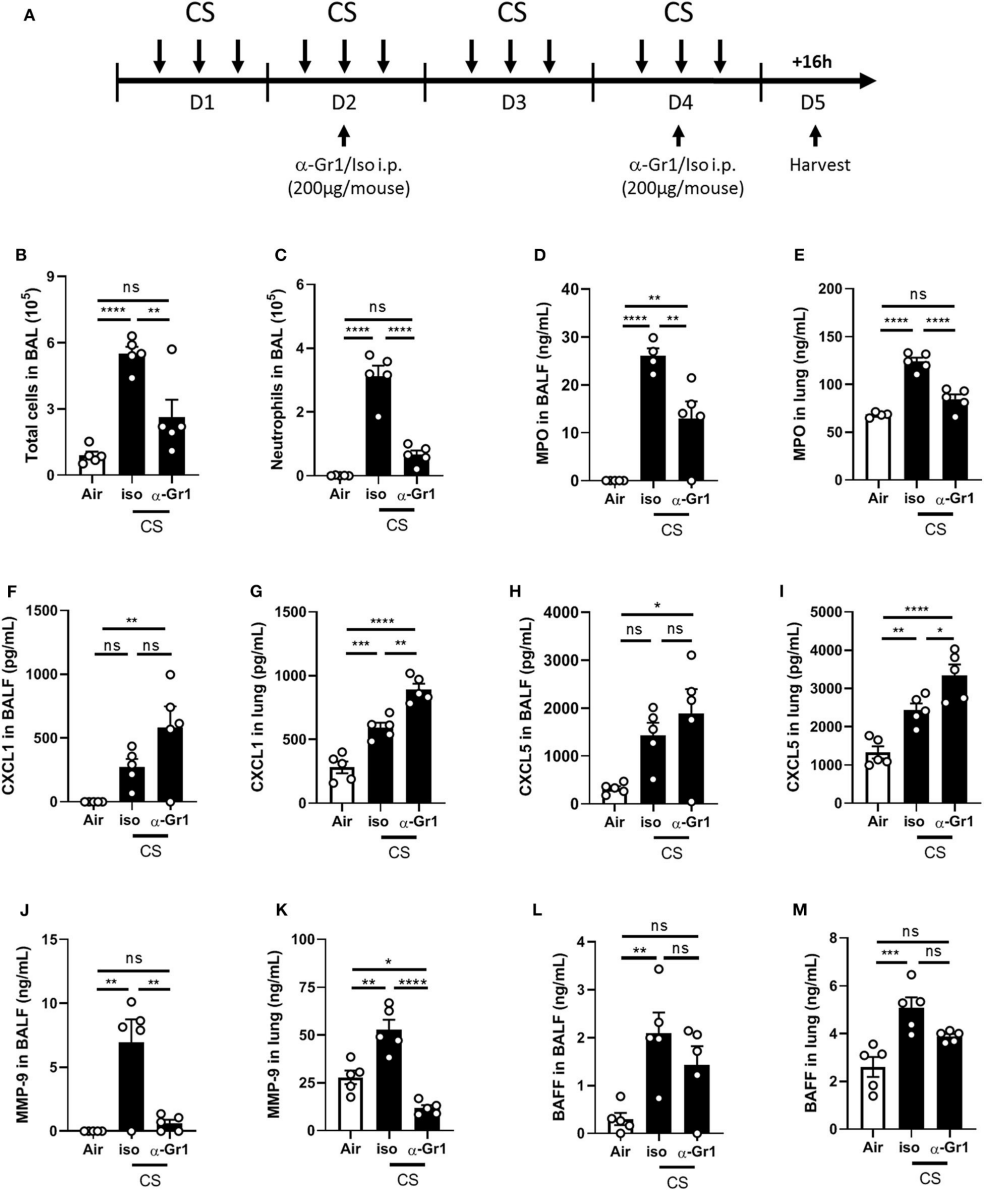
Neutrophil depletion partially decreases pulmonary BAFF levels upon CS-exposure. WT mice were exposed to air or CS. CS-exposed WT mice were injected intraperitoneally with 200 μg of anti-Gr1 or isotype control antidody per mouse at day 2 and 4 between the second and the third exposure **(A)**. Total cells **(B)** and neutrophils **(C)** counted in BAL. MPO **(D,E)**, CXCL1 **(F,G)**, CXCL5 **(H,I)**, MMP-9 **(J,K)**, and BAFF **(L,M)** levels were measured in BALF and lung, respectively. *n* = 4–5 mice per group. Bar graphs are expressed as mean ± SEM. ns, non-significant; *p < 0.05, **p < 0.01, ***p < 0.001, and ****p < 0.0001.

To validate the hypothesis that neutrophils are a major source of airway secreted BAFF upon mouse CS-exposure, we performed a neutrophil depletion experiment. Anti-Gr1 antibody (200 μg per mouse) or its isotype were injected intraperitoneally to WT mice at days 2 and 4, between the second and the third CS exposure (Figure 2A). Anti-Gr1 but not isotype control treated mice display decreased total cell number in BAL (Figure 2B) and in particular neutrophils (Figure 2C). However, neutrophil recruitment was not totally abolished indicating that neutrophil depletion was not complete. We also observed a significant decrease in MPO levels measured in BALF and lungs of anti-Gr1 treated mice as compared to isotype control treated mice (Figures 2D,E). Neutrophil-recruiting chemokines CXCL1 (Figures 2F,G) and CXCL5 (Figures 2H,I) levels were significantly increased in the lungs but not in the BALF of CS-exposed anti-Gr1-treated mice as compared to isotype control-treated mice. This increased CXCL1 and CXCL5 lung levels could be explained by the reduction of recruited neutrophils which express CXCR2 (the receptor for CXCL1 and CXCL5) and thus to an increase of unbound chemokines. In contrast, BALF and lung levels of matrix metalloproteinase-9 (MMP-9), known to be produced by neutrophils, were strongly decreased in CS-exposed mice treated within anti-Gr1 antibody (Figures 2J,K). Importantly BAFF production was significantly induced in BALF and lungs of CS-exposed isotype control-treated mice but not in CS-exposed anti-Gr1-treated mice suggesting an important contribution of neutrophils in BAFF production. However, we only observed a trend in the decrease of BAFF levels after anti-Gr1 antibody treatment in comparison to isotype control treatment (Figures 2L,M), probably due to incomplete neutrophil depletion or contribution of an additional cellular source of BAFF.

### *In vitro* CSE Stimulation Induces BAFF Expression in Neutrophils and Epithelial Cells

**Figure 3:**
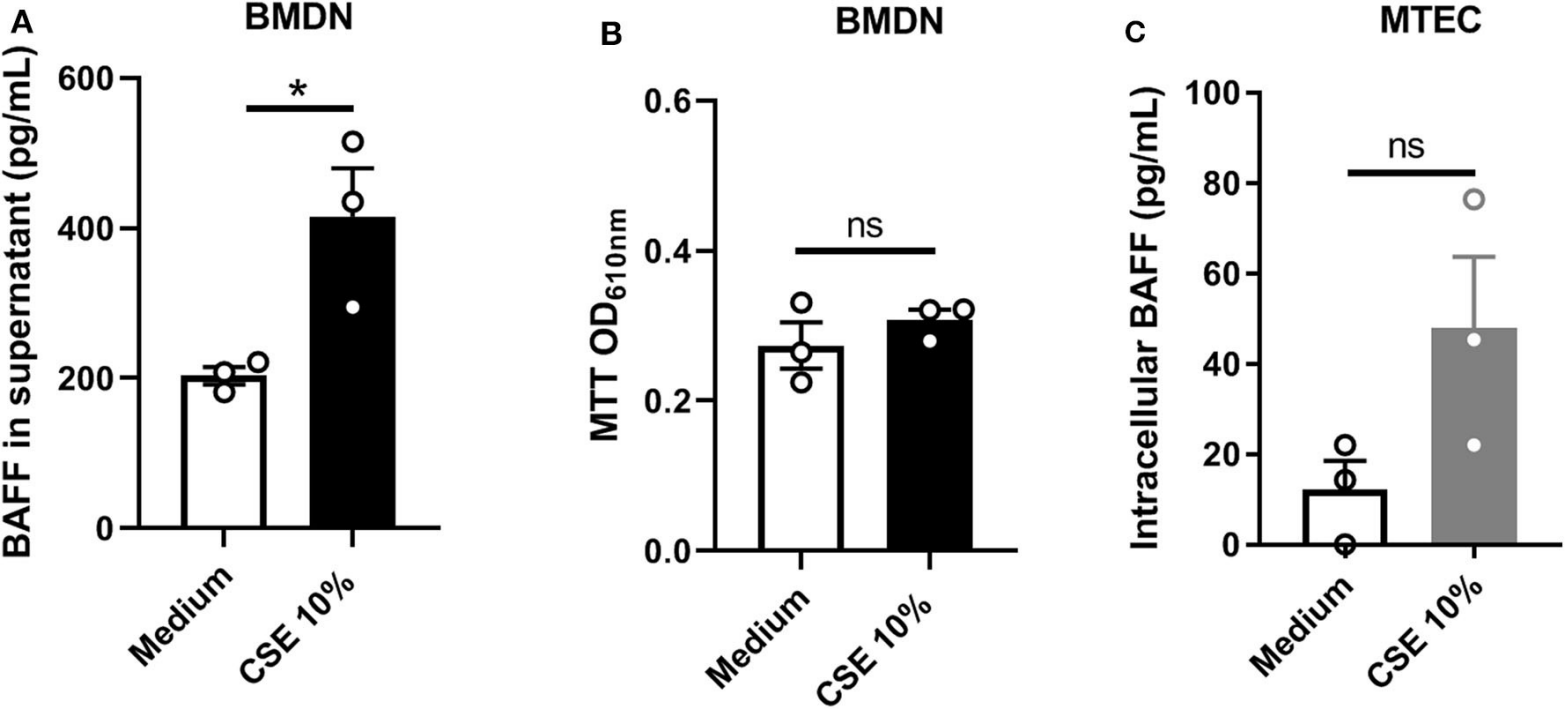
*In vitro* CSE stimulation induces BAFF expression in neutrophils and epithelial cells. Bone marrow derived neutrophils (BMDN) were stimulated with medium or medium containing 10% Cigarette Smoke Extrat (CSE) for 4 h. BAFF levels in supernatant **(A)** and MTT cell viability assay on cellular fraction **(B)**. Murine tracheal epithelial cells (MTEC) were stimulated with medium or medium containing 10% CSE. BAFF was measured in the cellular fraction **(C)**. Stimulation has been performed in triplicate for each condition. Bar graphs are expressed as mean ± SEM. ns, non-significant; *p < 0.05. Mann-Whitney *T*-test was performed.

To confirm that neutrophils can secrete BAFF, bone marrow-derived neutrophils (BMDN) were sorted and stimulated for 4 h with home-made 10% cigarette smoke extract (CSE). We observed that CSE stimulation significantly increased BAFF levels in the supernatant of BMDN isolated from WT mice, indicating that neutrophils are able to secrete BAFF in response to CSE (Figure 3A). At this time point, 10% CSE did not promote neutrophil cell death, as observed performing MTT assay (Figure 3B). Moreover, CSE stimulation of murine tracheal epithelial cells (MTEC) isolated from WT mice and maintained immersed *in vitro*, enhanced intracellular BAFF accumulation as compared to unstimulated MTEC (Figure 3C). However, BAFF was undetectable in the supernatant of CSE-stimulated MTEC suggesting an absence or a finely regulated BAFF secretion by these cells (data not shown). These results suggest that neutrophils rather than epithelial cells may be an important source of airway secreted BAFF in response to CS-exposure.

### BAFF Positively Regulates CS-Induced Airway Inflammation

**Figure 4:**
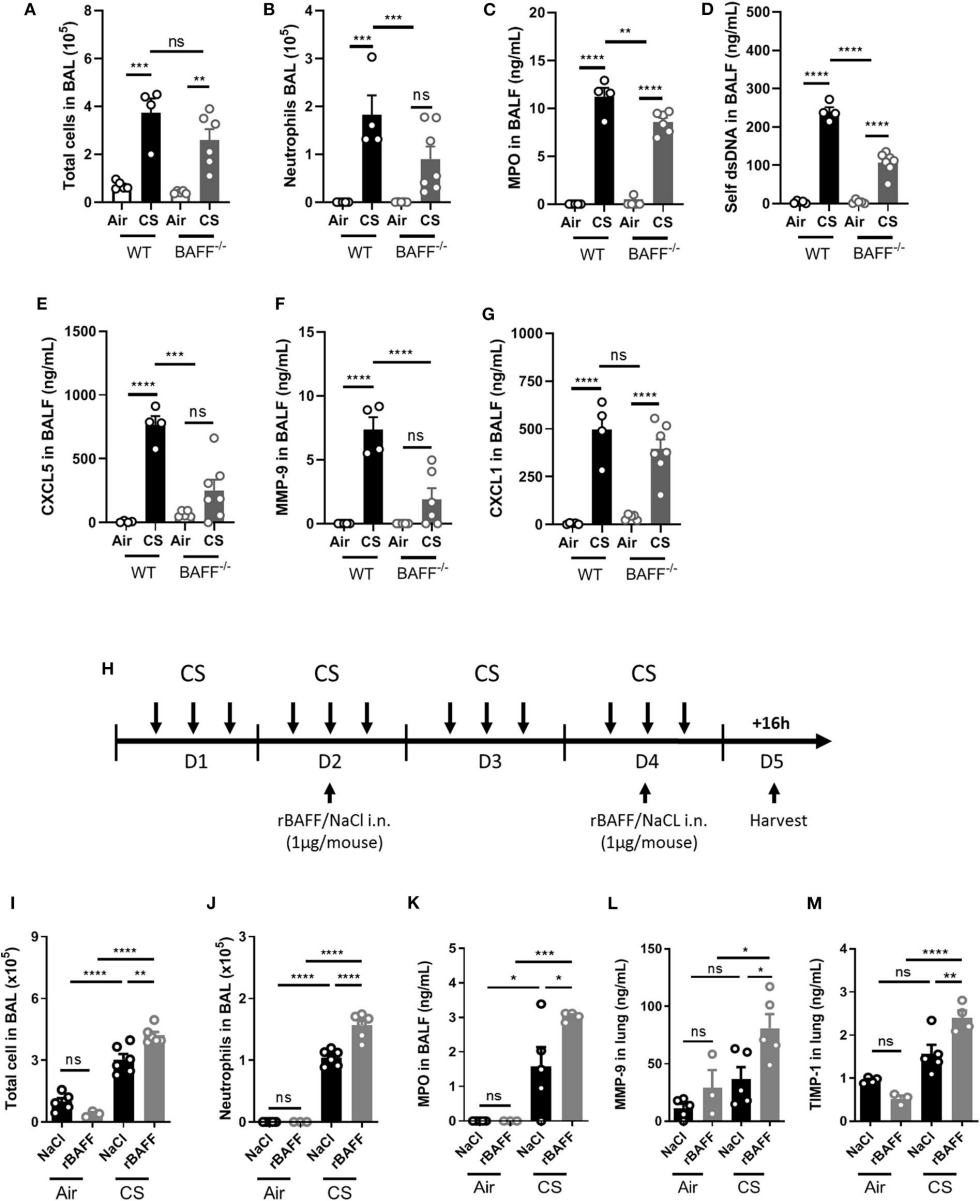
BAFF positively controls CS-induced inflammation. WT and BAFF deficient mice (BAFF^−/−^) were exposed to air or CS and sacrificed 16 h after the last exposure. WT mice were instillated with NaCl as control, or with rBAFF (1 μg/mouse) at days 2 and 4 between the second and the third exposure **(H)**. Total cells **(A,I)** and neutrophils **(B,J)** in BAL. MPO **(C,K)**, self-dsDNA **(D)**, CXCL5 **(E)**, CXCL1 **(G)**, MMP-9 **(F,L)**, and TIMP-1 **(M)** levels measured in BALF or lung as indicated. *n* = 3–7 mice per group. Bar graphs are expressed as mean ± SEM. ns, non-significant; *p < 0.05, **p < 0.01, ***p < 0.001, and ****p < 0.0001.

In order to study the role of BAFF in acute CS-induced lung inflammation, we exposed wild type (WT) and BAFF deficient mice (BAFF^−/−^) to air or CS. While total BAL cell numbers were not significantly decreased (Figure 4A), neutrophil numbers and MPO levels in the BAL were significantly decreased in CS-exposed BAFF^−/−^ mice as compared to WT mice (Figures 4B,C). Double-stranded (ds) self-DNA level in the BALF was also reduced in CS-exposed BAFF^−/−^ mice as compared to WT CS-exposed mice, suggesting lower CS-induced cell death and/or neutrophil extracellular traps (NETs) formation in these mice (Figure 4D). In addition, CXCL5 (Figure 4E) and MMP-9 (Figure 4F) BALF amounts were reduced in CS-exposed BAFF^−/−^ mice, whereas CXCL1 levels were not (Figure 4G).

We next intranasally administrated recombinant mouse BAFF (rBAFF) protein (1 μg/mouse) or NaCl at indicated time points (Figure 4H) and exposed mice to air or CS. CS-exposed rBAFF-treated mice presented a significant increase in BAL total cell (Figure 4I) and neutrophils (Figure 4J) in comparison to NaCl treated CS- or air-exposed mice. Higher neutrophil pulmonary influx observed in CS-exposed and rBAFF-treated mice was confirmed by increased BAL MPO levels (Figure 4K) that were significantly higher as compared to CS-exposed mice only. Moreover, remodeling factors in the lungs such as MMP-9 (Figure 4L) and TIMP-1 (Figure 4M) were also increased in CS-exposed rBAFF-treated mice in comparison to CS-exposed mice. In conclusion, BAFF deficiency resulted in decreased pulmonary inflammation to CS-exposure while the addition of exogenous BAFF to WT mice significantly enhanced inflammation. Altogether, these data show that BAFF is a key proinflammatory player during the acute phase of pulmonary inflammation induced by CS-exposure.

## Discussion

Here, we investigated whether BAFF is expressed during the early stages of cigarette smoke (CS)-induced inflammation and whether it participated in the development of the early pulmonary innate immune response. Previous works indicated that BAFF is strongly expressed in smokers and COPD patients leading to lymphoid follicles formation and pulmonary B cell survival (11, 20–23). In chronically CS-exposed mice, BAFF expression is greatly increased leading to the generation of pulmonary antinuclear antibodies and tertiary lymphoid tissues (11, 23). Another study reports that chronic exposure to cigarette smoke causes decreased airway BAFF levels leading to enhanced influenza virus infection (24). These discrepancies are unclear and might reflect the use of distinct mouse colonies and/or CS exposure protocol. Here we show that acute CS exposure promotes BAFF expression in the bronchoalveolar space. Early CS-induced expression of BAFF highlights BAFF as a crucial mediator in the crosstalk between innate and adaptive immune responses in the establishment of pulmonary chronic inflammation.

We previously identified Gr1^+^ neutrophils as an important source of BAFF upon BLM-induced lung inflammation and fibrosis (10). Here, we show in our model that airway neutrophils are the main BAFF producing cells in the bronchoalveolar space suggesting that recruited neutrophils are the major source of BAFF during acute pulmonary inflammation to CS. In contrast, one study showed that some lung tissue resident alveolar macrophages express BAFF upon mice acute CS-exposure and proposed that macrophages are the essential source of pulmonary BAFF (11). We cannot exclude that macrophages may also produce BAFF, in particular lung tissue macrophages as observed by these authors.

Neutrophil depletion experiment confirmed that neutrophils are important inducers of early pulmonary inflammation. Neutrophil depletion partially decreases BAFF expression in lung tissue and bronchoalveolar space of CS-exposed mice suggesting that while neutrophils are important BAFF producers, additional sources exist, possibly alveolar macrophages (11) or reflecting incomplete neutrophil depletion.

Exploring this hypothesis, we showed that murine bone marrow-derived neutrophils stimulated *in vitro* with cigarette smoke extract (CSE) secrete BAFF, confirming that neutrophils are one of the main source of BAFF. Moreover, epithelial cells also expressed BAFF after CSE stimulation, but did not secrete it in this experimental setting. This observation suggests that epithelial cells could be, *in vivo*, another source of BAFF during CS-induced lung inflammation. BAFF-containing epithelial cells could explain the presence of airway BAFF in air-exposed mice that do not present airway neutrophils. In addition, a recent study showed that BAFF production may be induced in a mouse macrophage cell line by hydrogen peroxide, an oxidant generated following CS exposure (25). However, in contrast to bone marrow-derived neutrophils, we did not observed BAFF production by CSE-stimulated bone marrow-derived macrophages.

Of note, the use of different exposure models (26), different mouse strain or sex (25) change the response observed (27).

Proinflammatory mediators were shown to elicit secretion of the intracellular or surface expressed BAFF during pathologic inflammatory responses (16, 28). BAFF was associated with autoimmunity and recent evidence suggests that autoimmune processes are involved in the pathogenesis of COPD (29). In this context, neutrophils were shown to produce BAFF and contribute to excess serum BAFF levels, promoting CD4^+^ T cell and B cell responses in autoimmunity model of lupus-prone mice (30). In addition, prolonged infections or adjuvant usage can trigger emergency granulopoiesis, leading to dysregulated neutrophil blood counts. The recruited neutrophils secreted BAFF that highly accelerated plasma cell generation and antigen-specific antibody (31).

Importantly, we showed that neutrophil and myeloperoxidase level reduction in the airways was associated with decreased airway self-dsDNA content in BAFF deficient mice. These results indicate that BAFF is necessary for neutrophilic inflammation and suggest that neutrophils may release self-DNA through NETs formation which are released during NETosis cell death. NETs are web-like scaffolds of extracellular DNA in complex with histones and neutrophil granular proteins, such as myeloperoxidase and neutrophil elastase. Interestingly, NETopathic inflammation was recently implicated in the pathogenesis of COPD (11, 32, 33).

In addition recent studies showed that autoinflammatory NETs are composed of self-DNA, neutrophil elastase autoantigen but also BAFF protein, inducing anti-dsDNA antibodies (34, 35). It will be interesting to analyze whether neutrophil-associated BAFF release upon CS exposure is occurring through active BAFF secretion or NETosis. In lungs of patients with COPD, BAFF expression was increased in immune cells, in particular alveolar macrophages, but also in stromal cells in lymphoid follicles (20, 23). BAFF expressing cells may differ depending on the chronicity of inflammation and/or on COPD stage.

Finally, we report a critical role of BAFF in acute inflammation to CS in mice since genetic ablation of BAFF significantly attenuated airway neutrophilic inflammation. In the same way, BAFFR-Fc administration was shown to significantly attenuate lung inflammation and alveolar wall destruction confirming that BAFF has proinflammatory effect (23). Addition of fairly large amounts of recombinant BAFF to observe whether its effects are additive to those mediated by CS-induced BAFF confirms that BAFF positively regulates airway neutrophilic lung inflammation. It is worth mentioning that 1 μg recombinant BAFF administration to naïve mice did not lead to significant differences in terms of BAL cell recruitment and remodeling factors production. Since neutrophils were not showed to express BAFF receptors, these results suggest that additional mediators are required. We previously showed that CS exposure induces IL-1β production, which is important to promote neutrophil recruitment as we previously showed (36). Other demonstrated the role of IL-17 in neutrophil influx in response to CS exposure (37, 38). One hypothesis is that BAFF may induces IL-1β and/or IL-17 production in a feedback loop, leading to neutrophil influx. Since we did not observe an increase in *Tnfsf13* expression, it appears that among the TNF family, BAFF but not APRIL plays a major role in CS-induced inflammation. In conclusion, our data demonstrate that innate immune cells and in particular neutrophils are an important source of BAFF in early stages of CS-induced lung inflammation and suggest that BAFF is a crucial mediator in the crosstalk between innate and adaptive immune responses upon cigarette smoke exposure.

## Materials and Methods

### Mice

Eight to twelve weeks old wild-type C57BL/6J (WT) male mice were purchased from the Janvier laboratory (Janvier Laboratory, France). BAFF^−/−^ (39) were provided by Pascal Schneider from Lausanne University. All mice were backcrossed 10 times on C57BL/6J background and housed in the UPS44-TAAM (CNRS, Orleans, France) animal facility. For experiments, adults males (8–12 weeks old) were kept in sterile, isolated and ventilated cages.

### Cigarette Smoke Model

CS exposure was performed using a calibrated EMKA InExpose smoking robot. Mice were exposed to mainstream cigarette smoke in a whole-body chamber for 20 min, 3 times per day for 4 days. We used 3R4F research cigarettes (University of Kentucky) with the filter removed and the cigarettes were puffed once per minute, 4 s duration, 200 ml puff volume. The experimental bias flow, required to deliver CS and fresh air to the mice, is calibrated at 3.107 L.min-1 and maintained constant. We did not measure the concentration of smoke particulates, but the manufacturer estimates the exposures to be 350 mg/cubic meter.

### Treatment

Mice were treated intraperitoneally with 200 μg of mouse anti-Gr1 (BE0075, Euromedex) or isotype control (BE0090, Euromedex) at day 2 and 4 between the second and the third exposition. Recombinant mouse BAFF (8876-BF-010, R&D Systems) was administred intranasally (1 μg/mouse) at days 2 and 4 of CS exposure between the second and the third daily exposure. Mice were anesthetized using intramuscular injection of ketamine/xylasine.

### Broncho-Alveolar Lavage (BAL)

BAL was performed as previously described (40). Bronchoalveolar lavage (BAL) and lung tissue were harvested 16 h after the last CS exposition. Differential cell counts were performed by counting an average of 250 cells on Cytospin preparations (Shandon CytoSpin3, Thermo Scientific) after May-Grünwald-Giemsa (MGG) staining (Diff Quick, Medion Diagnostics) according to manufacturer's instructions.

### Lung Homogenates

After BAL the lungs were perfused with Isoton® (Beckman Coulter France, Villepinte) to flush the vascular content. Lungs were homogenized by a rotor-stator (Ultra-turrax®) in 1 ml of PBS for ELISA dosage. The extract was centrifuged 10 min 10,000 rpm and the supernatant was stored at −80°C before mediator measurement.

### Mediator Measurements

For cytokine determination, BALF supernatant and lung homogenates or culture supernatant were analyzed by ELISA assay kits for murine: CXCL1, CXCL5, MPO, MMP-9, TIMP-1, and BAFF (R&D system) according to manufacturer's instructions.

### Measurement of Double-Stranded DNA

Double-stranded DNA was measured in the BAL fluid (BALF) using Quant-iTPicoGreen dsDNA reagent (Invitrogen, Carlsbad, CA), according to the manufacturer's protocol.

### Quantitative RT-PCR

RNA was purified from lung homogenates by using Tri-Reagent (Sigma-Aldrich) extraction protocol. Reverse transcription of RNA into cDNA was carried out with GoScript™ Reverse Transcription System (Promega). RT-qPCR was performed with Fast SYBR Green Master mix (Promega) on anARIAMX(Agilent Technologies). Primers for *Tnfsf13 (*#QT00254023) were purchased from Qiagen (Qiagen, Hilden, Germany). RNA expression was normalized to *Gapdh* (#QT00166768) expression and analyzed using the ^ΔΔ^Ct method.

### Immunostaining on Cytospin

Cytospin slides were fixed in paraformaldehyde 4% (Sigma-Aldrich). After 3 lavages in TBS, cells were incubated 10 min in TBS-0.3% Triton X-100, were washed 3 times in TBS, blocked in TBS-1% BSA-10% SVF during 45 min and incubated with primary anti-BAFF (Abcam, ab16081) or control isotype antibodies over night at 4°C. After 3 lavages in TBS, cells were incubated 1 h at room temperature with anti-rat IgM conjugated to Alexa 488. Cytospins were counterstained using 4′,6-diamidino-2-phenylindole (DAPI) for 10 min, rinsed and coverslip were mounted with Mowiol (Sigma-Aldrich). For fluorescent intensity quantification, the gray value was calculated in each cell selected on untreated pictures. Cells were observed using a Nikon eclipse 80i microscope and images were treated using ImageJ software.

### Bone Marrow Derived Neutrophils

Femurs from 7 weeks old WT mice were collected and rapidly flushed in sterile conditions with RPMI/10% FBS and smashed through a 100 μm filter to remove aggregates and centrifuge at 1,400 rpm for 5 min. The pellet was resuspended in PharmLyse solution (BD Biosciences) to lyse red blood cells. Lysis reaction was stopped by adding FBS and then centrifuged 5 min at 1,400 rpm. Pellet was resuspended in PBS-FBS 0.5%-EDTA 2 mM buffer prior to isolation. Neutrophils were isolated using the Neutrophil Isolation Kit (MiltenyiBiotec) following manufacturer instructions. Briefly, cells were stained with a cocktail of biotin-conjugated monoclonal antibodies not expressed on neutrophils. After that, cells were stained with magnetic anti-biotin antibodies and separated using magnetic LS Columns (MiltenyiBiotec). Cells unretained by the colums were numerated and seeded in a 96 well-plate at 1.10^5^ cells per well prior to stimulation. Supernatant was collected after 4 h stimulation and stored at −80°C for further analysis. Cell death was monitored by MTT using a standard protocol. Thiazollyl blue tetrazolium bromide (Sigma) solution was added onto the cells after supernatant collection and incubated for 2 h at 37°C, and a 10% SDS acetic acid solution was then added. MTT reduction to formazan was quantified by an absorbance microplate reader (EL800, BioTek, Colmar) at 610 nm (KC4 software).

### Murine Tracheal Epithelial Cells

Trachea from WT mice were collected in DMEM-F12 GlutaMAX medium (Thermofisher Scientific) containing Penicillin-Streptomycin (100 U/mL, Fisher) cocktail and incubated over night at 4°C with Pronase (1.5 mg/mL, Sigma-Aldrich). Cells were collected and washed 3 times with DMEM-F12 GlutaMAX-10% FBS, 100 μm filtered, and centrifuged at 1,500 rpm for 10 min. Cell pellet was resuspended in DNAse solution (0.5 mg/mL, Sigma-Aldrich) for 5 min. Suspension was centrifuged for 10 min at 1,500 rpm and the pellet was resuspended in DMEM-F12 GlutaMAX media and incubated in Primaria plates (353801, VWR) at 37°C for 4 h to remove potential fibroblasts. Unadherent cells were then collected, centrifuged at 1,500 rpm for 10 min, counted and plated in 24 well-plate coated with rat tail collagen (ThermoFisher Scientific) at 50 000 cells per well. At this step, DMEM-F12 GlutaMAX-5% FBS medium containing Insulin-Transferrin (10 μM ThermoFisher Scientific), Cholera Toxin (0.10 μg/mL, Sigma-Aldrich) Epidermal Growth Factor (0.025 μg/mL, Sigma-Aldrich), Bovine Pituitary Extract (30 μg/mL, Fisher), and Retinoic acid (50 nM, Sigma-Aldrich) was used to maintain epithelial cells prior to stimulation. Supernatant and intracellular fraction were stored at −80°C for further analysis.

### *In vitro* Cell Stimulation

Cigarette Smoke Extract was prepared by bubbling the smoke of six 3R4F cigarettes with filter per 100 mL of media. This solution is considered as 100% CSE medium stock solution.

### Statistical Analysis

Statistical evaluation of differences between experimental groups was determined by Mann Whitney *T*-Test or one-way ANOVA, analysis of variance, Bonferroni test as indicated using GraphPad Prism software v.8. *P* <0.05 were considered statistically significant. All figures are representative of at least two different experiments.

## Data Availability Statement

The raw data supporting the conclusions of this article will be made available by the authors, without undue reservation.

## Ethics Statement

The animal study was reviewed and approved by Ethics Committee for Animal Experimentation of CNRS Campus Orleans (CCO) under number CLE CCO 2015-1088.

## Author Contributions

MN, MB, AG, CP, SH-M, NR, MF, and FS performed the experiments. MN, AG, MLB, and IC conceived the experiments and analyzed the data. MN and MLB supervised the breeding of knock-out mice. MN, AG, NR, MLB, BR, VQ, and IC discussed the results. PS provided the BAFF^−/−^ mice. MN, AG, NR, and IC prepared the paper. IC and VQ provided funding. IC overall supervision of of this study. All authors contributed to the article and approved the submitted version.

## Conflict of Interest

The authors declare that the research was conducted in the absence of any commercial or financial relationships that could be construed as a potential conflict of interest.

## Abbreviations

CXCL: C-X-C motif chemokine
CS: cigarette smoke
COPD: chronic obstructive pulmonary disease
BAFF: B cell activating factor
BAL: bronchoalveolar lavage
BALF: BAL fluid.
